# Valorize to Protect Lucanian Sheep and Goat Biodiversity

**DOI:** 10.3390/ani12070884

**Published:** 2022-03-31

**Authors:** Maria Assunta D’Oronzio, Carmela De Vivo, Teresa Lettieri, Paola Telesca, Milena Verrascina

**Affiliations:** 1 CREA, Council for Agricultural Research and Agricultural Economics Analysis Research Center for Politics and Bioeconomy, 85100 Potenza, Italy; carmela.devivo@crea.gov.it (C.D.V.); teresa.lettieri@crea.gov.it (T.L.); paola.telesca@crea.gov.it (P.T.); 2 CREA, Council for Agricultural Research and Agricultural Economics Analysis Research Center for Politics and Bioeconomy, 00198 Roma, Italy; milena.verrascina@crea.gov.it

**Keywords:** consumer attitudes, biodiversity, traditional and quality food, sustainability

## Abstract

**Simple Summary:**

Autochthonous sheep and goat breeds are at risk of extinction as they produce less milk and meat compared to other breeds, around 50% less for milk and 30% less for meat production, leading to a reduction in the number of animals bred. However, thanks to rural policies, sheep and goat biodiversity is recovering and livestock farms can recognize and sustain their added value to the territory, the landscape, the maintenance of biodiversity and to sustainability through the adoption of a local supply chain in the medium and long term. Financial support for farms is not enough, integrated policies are needed to focus on further training and education and to improve communication channels to increase consumer awareness of biodiversity and quality.

**Abstract:**

European agriculture and rural development policies have promoted the diversity and genetic types of autochthonous breeds to increase sheep and goat biodiversity. Agri-environmental measures to support livestock farmers, have been the main tools used by these policies over the last twenty years. The COLAUTOC, “Collection of a seed bank for native sheep and goat breeds and strategies to increase their numbers”, research project in Basilicata, Southern Italy, investigated sheep and goat farms with autochthonous breeds with results proving a reduction in the number of sheep and goat farms in general, particularly those with native breeds at risk of extinction, a clear indication of policy failure with a devastating loss of sheep and goat biodiversity. The COLAUTOC used a variety of methodological tools, including, desk analysis, focus groups, interviews, school workshops and a survey on sheep and goat meat consumption. The results indicated that a recovery in sheep and goat biodiversity could be achieved by supporting farm livestock strategies with a view to diversify production methods and activities, whilst, recognizing the value added to the territory, landscape, biodiversity and sustainability. The strategies identified by the COLAUTOC project require a local vision, using agri-environmental funds to invest in training on innovation for farms, provide tools to improve the quality of life in the rural areas, to increase communication channels to further knowledge and awareness of biodiversity. These strategies could contribute to meeting several 2030 Agenda goals.

## 1. Introduction

Increasing the number of endangered native sheep and goats in Basilicata is crucial for the development of sustainable biodiversity from an ecological, economic and social perspective. The local agro-pastoral system [1] fits the adaptive cycles [2,3,4] of exploitation, conservation, collapse and reorganization, determined by a wide and articulated network of interactions between people and the surrounding environment. The resilient [5] characteristics of animal husbandry requires a reorganization of the ecological available resources to enhance marginal and inner areas, guaranteeing the production of consumer goods and services, while preserving public assets such as landscape, environmental and social biodiversity, animal welfare, quality of life, cultural traditions and heritage [6]. As a result, the sheep and goat sector provides “benefits” to the local economy [7,8,9].

Sector comparison and literature analyses refer to “adaptive governance” as the most effective coordination tool, based on networks of individuals, agencies and institutions at various organizational levels, who are capable of undertaking progressive or new directional changes to improve structures and processes to create individual and collective actions [10]. The conservation of biodiversity is an essential part of ecosystems and it is necessary to introduce agricultural multifunctionality [11,12,13] to improve income opportunity.

Recent research shows that various livestock farms consider agricultural multifunctionality as essential in the generation of short supply chains [14], direct relationships [15] and collective benefits. Agricultural multifunctionality also maximizes labor returns and boosts ecological capital, tightly tying corporate income to the territory and reducing its dependence on the global market [16].

A new European agricultural model is emerging, centered on agronomically sound and sustainable agricultural systems, characterized by high-added-value farming and high-quality primary and processed products [17]. The new agricultural strategies are quality differentiation and the construction of new alternative markets where high quality products meet consumers needs [18].

There are more than 70 million sheep and goats (85% sheep and 15% goats) in the EU, often raised in economically fragile areas such as mountainous regions [19]. 

Goat and sheep farming systems in the Mediterranean represent one of the most important agricultural activities, particularly in fragile inner areas with more prevalent pastoral systems, including low levels of mechanization and the production of typical products, mainly cheeses [20]. 

The sheep and goat supply chain in Italy has little economic relevance compared to national agricultural production with an Added Value (VA) of just over 1% of the national agriculture VA [21]. The Italian sheep and goat sector is mainly oriented towards dairy production while meat is a secondary product that generates a value equal to one third of that of sheep’s milk (163 million for meat and 442 million for milk) [21].

The national sheep and goat population amounts to 6,179,121 sheep and 1,054,549 goats [22]; stable over the last five years. 

Sheep and goat meat consumption is concentrated almost exclusively in two periods of the year coinciding with the Easter and Christmas holidays, with a legacy of traditions that are still very much alive in society. In recent years, the meat sector has been facing a crisis linked to the effects of an increase in vegetarian and vegan diets. The closure of the HO.RE.CA channels, the prolonged absence of tourists and the restrictions due to the COVID-19 pandemic have compromised the lamb market.

The EU rural development policy links a territorial approach to specific growth objectives through the integration of tools, resources and interventions [23]. 

Farm diversification and multifunctionality hold a place in the European rural development policy as they are considered a lever of socio-economic development that addresses the typical rural area problems such as an ageing population, declining employment, rural exodus, preservation of territory and its rural heritage, availability of services, new business activities, income sources, quality products and the environment [24]. 

The COLAUTOC project, “Collection of a seed bank for native sheep and goat breeds and strategies to increase their numbers”, was approved in 2017 and financed by the 2014–2020 Basilicata RDP—Sub-measure 10.2 “support for the conservation of genetic resources in agriculture and forestry”. The goals include the protection and enhancement of four endangered sheep breeds (Gentile di Puglia, Leccese, Altamurana and Trimeticcio di Segezia) and four native endangered goat breeds (Capra di Potenza, Garganica, Jonica, Rossa Mediterranea).

The aim of this paper is the identification of possible development paths linked to the territory, landscape, biodiversity and sustainability that could be supported by public policy through the multifunctional approach.

## 2. Materials and Methods

Over the last twenty years, the animal husbandry sector has seen the progressive replacement of both prestigious and specialized native and rustic breeds. With so many indigenous breeds at risk of extinction, serious socio-economic repercussions would impact the protection of the territory. Indeed, beyond their purely productive function, sheep and goat farms maintain a strategic role in the environmental landscape and cultural protection of the hilly mountainous areas, particularly those that are marginalized. 

**Figure 1:**
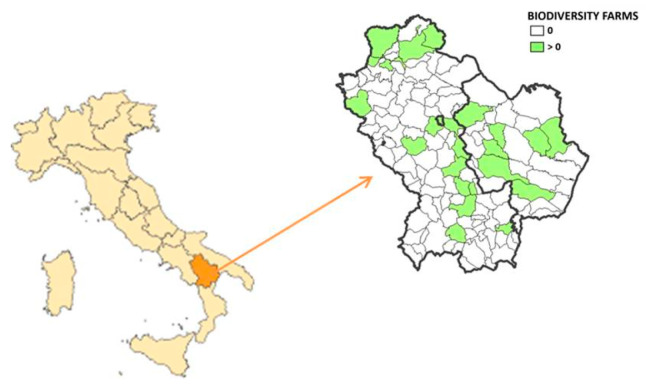
Localization of Lucanian biodiversity farms, Basilicata, Italy. Source: CREA PB (2021).

Basilicata is rich in vegetal and animal agricultural biodiversity with close links to cultural and gastronomic traditions (Figure 1).

The protection of biodiversity is fundamental in the maintenance of a natural balance. The data show 42,078 raised goats and 171,536 raised sheep in Basilicata [22] with the number of raised animals in continuous decline. 

**Figure 2:**
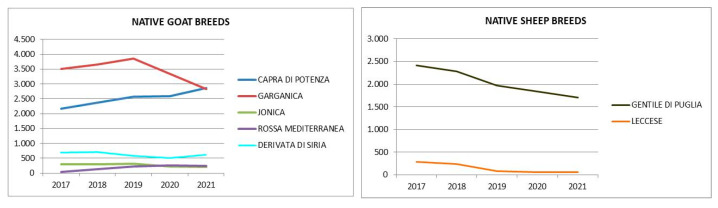
The 2021–2017 native Lucanian sheep and goat breed population trends (No). Source: National Zootechnical Register, Statistics, elaborated by CREA PB.

The trend in the population of native sheep and goat breeds has reduced in the past 5 years (Figure 2), with the exception of the Capra di Potenza and the Rossa Mediterranea breeds which have increased.

Sheep and goat production is 2.2% of the total Lucanian agriculture, including dairy products [25], in particular “Pecorino di Filiano” and “Canestrato di Moliterno”, two cheeses with a protected designation of origin (PDO), and a range of cheeses which are traditional agricultural products (PAT) [26], such as Cacioricotta, Caprino, Casieddo, Pedraccio, Pecorino and mixed Pecorino.

In an attempt to promote its popularity in 2008, the “Agnello delle Dolomiti Lucane” brand [27] (product recognized in the P.A.T. list) was launched, it is the property of the Edere Lucanum Cooperative, which was created to promote local goat and sheep production using a local supply chain and ensuring consumer safety. The cooperative brings together livestock farms from the Lucanian hills and mountains dedicated to the breeding of sheep and goats and has launched an integrated supply chain project for the enhancement of lambs and kids born and raised in the mountain areas [27].

The EU plays an active role in the protection of biodiversity and in October 2020, member states approved the 2030 targets proposed by the Commission to step up efforts to protect and restore the natural environment and ecosystems. At the international level, the EU contributes to ensuring compliance to its global commitments to protect nature and biodiversity, through multilateral conventions such as the Convention on Biological Diversity and the Convention on International Trade concerning endangered species of wild fauna and flora. 

These strategies have been applied through community planning for agriculture and rural development, in the provision of specific measures of the RDPs aimed at biodiversity, dedicating funding to preserve, restore and enhance ecosystems. The 2007–2013 Basilicata RDP [28] has already considered measures to protect the environment from the promotion of organic agriculture to the conservation of endangered breeds and the provision of indemnities linked to the presence of farms in sites of particular environmental value [29] (Natura 2000 sites) and in areas subject to natural constraints. In particular, Measure 214 dedicated to the subject matter of animal biodiversity safety with: Action 3—Sub-Action B “Protect and conserve local animal breeds in danger of extinction”. This measure protected the endangered breeds through actions supporting conservation interventions, mainly in situ, of native breeds. Action 5 “Agrobiodiversity—Integrated action projects” to encourage the implementation, recovery and improvement of agrobiodiversity. It should be noted that these actions have not received much attention, with only 53 farms benefiting from Action 3 [30] and eleven projects approved for Action 5 [31]. The reasons could be due to the lack of information and assistance to farmers on the opportunities of the tenders, and in the low amounts of aid which are not particularly desirable incentives.

The Basilicata region [32] has allocated 43% of financial resources to biodiversity and environmental sustainability objectives from the 2014–2020 agricultural programme, using further actions to promote the collection, characterization, use, conservation and enhancement of animal genetic resources, particularly through two sub-measures: 10.1.3 “Breeders and Growers Custodians” and 10.2 “Conservation and Sustainable Use of Genetic Resources in Agriculture”. 

The first sub-measure supports the protection of animal breeds and vegetal genetic diversity registered in the Regional Directory for the protection of autochthonous vegetal and animal genetic resources of agricultural interest. The Directory was established by Regional Law no.26 of 2008 [33] with the aim of maintaining and increasing consistency through on-site breeding, favoring a livestock production regime capable of guaranteeing high-quality production and recognized its added value [34,35,36]. Participation of farms in the sub-measure during this period is low, despite the presence of a network of custodian farmers and germplasm banks that house many autochthonous breed seeds which are of agricultural interest and are at risk of extinction.

The second sub-measure funded native breed farms in partnership with the Research Body Institute; eleven projects including 149 partners were funded [37]. 

Increasing the number of native breeds is, without a doubt, the best strategy for conserving biodiversity, and is also useful in the response to environmental sustainability and the evolution of consumer demand, as well as contributing to the security of farms in the area [20,38].

Various research tools were adopted including field surveys and desk analysis, the latter focusing on national and regional data and the results were shared with regional experts from the sheep and goat sector (production, research and other institutions) at two focus groups.

The Regional Breeders Association (A.R.A., project partner) gave us the 34 Lucanian biodiversity farms with local breeds of interest, that is all the goat and sheep regional biodiversity farms. Authors interviewed the 34 farms over the telephone between May and June 2020, based on the following:

☞ F The figure of the farmer and the role of his family on the farm;
☞ Type and characteristics of sheep and goat breeding;
☞ Production and marketing of the products;
☞ Quality of production and/or presence of a farm brand;
☞ The use of technology;
☞ Farm land infrastructure;
☞ Collaboration in business activities with third parties.

A second survey was carried out online with over 650 responses on the daily purchasing and consumption habits of Lucanian sheep and goat meat in Italy also using official Ministry of Agricultural Policy channels and social platforms.

In addition, one study visit was carried out by classes from Year Four, from two schools (an Agrarian Institute and an Arts High School), called “rural walks”, in order observe the biodiversity farm. Students met farmers on their land who provided them with first-hand experience of their sheep and goat biodiversity farms. 

Workshops were carried out in both schools over the following months. 

## 3. Results

**Figure 3:**
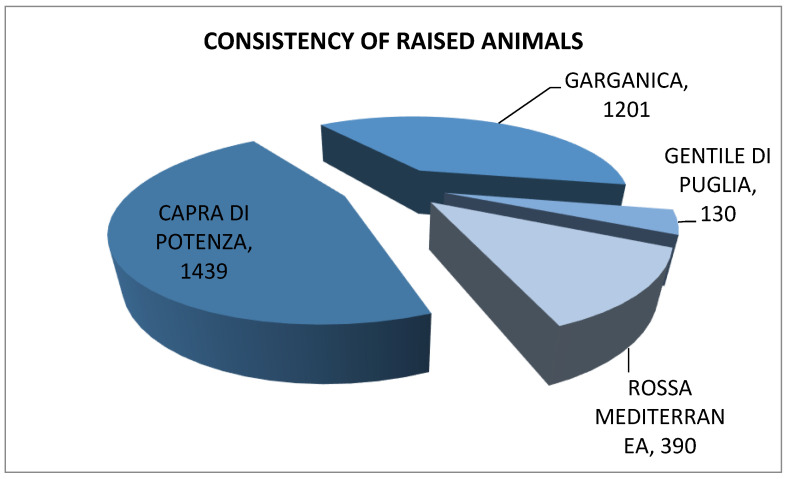
Consistency of raised animals (No.). Source: CREA PB—Biodiversity Farms (2020).

Analysis of local native sheep and goat farms in Lucania (Figure 3) found that 46% raise the “Capra di Potenza”, 38% the “Garganica” goat, with the “Rossa Mediterranea” goat and the “Gentile di Puglia” sheep taking up the remaining 16%. Direct business management is the most widely used method adopted by family-run businesses, with sheep and goats raised in extensive systems, mainly fed on Lucanian pastures. Product quality is the main strength of food production in these areas and should be recognized as adding value.

The autochthonous breeds of the COLAUTOC project compared to other breeds raised in Basilicata are characterized by lower yields of milk and meat. The difference is significant, about 50% less for milk production and 30% less for meat production. The low productivity of biodiversity breeds has, over time, led to a reduction in the number of animals bred [34,39,40].

**Figure 4:**
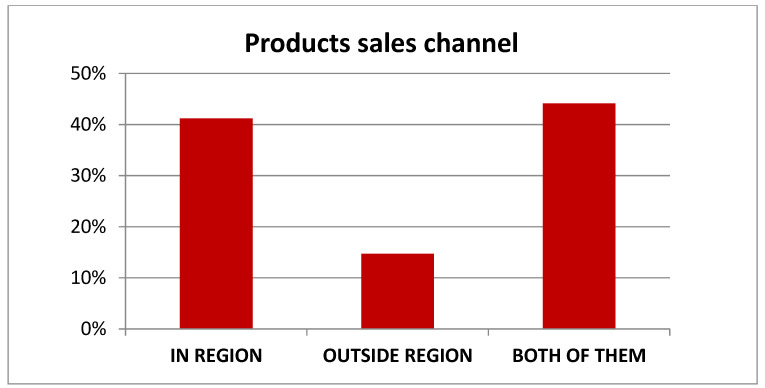
Products sales channel. Source: CREA PB—Biodiversity Farms (2020).

In total, 44% of farmers sell the raw material (meat and/or milk) to wholesalers or processing cooperatives in regional and national territories (Figure 4). 

Only 9% of breeders support the “Agnello delle Dolomiti Lucane” brand which was created with the aim of ensuring a better position on the market and to recognize the quality linked to the territory. The marketing methods are often not efficient or effective.

The use of technological devices to carry out farm activities was detected in 79% of farms; 38% of the interviewees used a company computer and 41% used a smartphone.

Farmers found that municipal and/or provincial road infrastructures were difficult to use due to their state of repair, even more complicated by the restrictive access routes to farms, classified as “bad” by 47% of farmers; some farmers are forced to reach farms exclusively on foot in adverse weather conditions. In addition, biodiversity farms have been heavily affected by the COVID-19 pandemic and the economic crisis of the last two years. In fact, the farms interviewed highlighted the need for training and consultancy services capable of supporting breeding activities, products and marketing methods.

The second survey identified consumer purchasing tendencies, providing a comprehensive review of the future prospects of the sector and potential solutions to reverse the disappearance trend, particularly in indigenous breeding.

**Figure 5:**
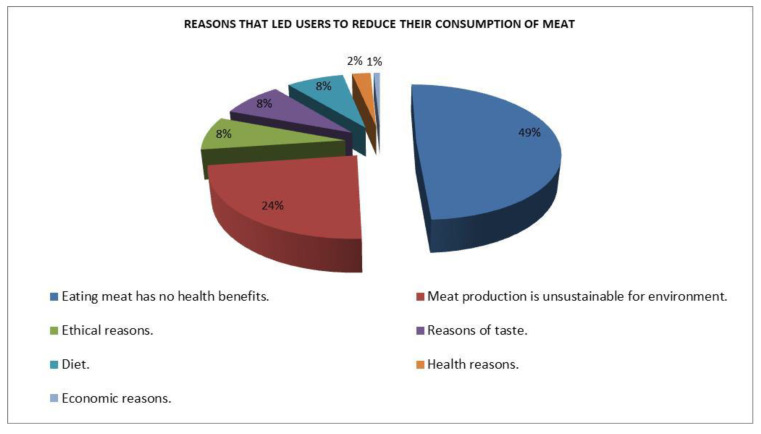
Reasons for reducing meat consumption. Source: CREA PB—Meat Consumption Survey (April 2021).

The survey highlighted a growing sensitivity to issues such as production sustainability and animal welfare that influences eating habits and purchasing choices. Although 49% of the sample stated that they have reduced meat consumption in recent years as they are convinced that it does not bring health benefits, the remainder often cited environmental and ethical aspects as a reason for a reduction in meat consumption (Figure 5).

In total, 33% of the interviewees said they would still be willing to buy more meat if it was from local agro-zootechnical farms (short chain) or if they were sure of the traceability of the product. Improving the quality of products and the environment, through less use of preservatives, buying seasonal produce, reducing pollution from transport, etc. and the exploitation of the features of the territory are considered essential in the promotion of the growth of the local economy.

The third activity was aimed at informing young people of the importance of local goat and sheep breeds and their related products, the landscape, biodiversity and sustainability and the imminent threat to local traditions handed down from generation to generation. Two high schools were chosen to take part in school study visits of the farms and then attended workshops, the Agrarian Institute and the Artistic Institute (Arts, Music and Dance). Students interviewed a farmer and his family and gathered photographic material and during the workshops, developed a business plan and a brochure with a logo and painted scenes depicting their experience. The farm was representative of the local mountains, types of pastures and animals and the “physical” differences between varieties and breeds. 

The results from desk analyses, focus groups and exchanges with students highlighted that the sector would benefit from an in-depth knowledge of the product quality, production methods and ethical aspects linked to social and environmental sustainability which are little known to the public. 

## 4. Discussion

The Lucanian sheep and goat sector has two different facets; on the one hand, biodiversity farms with a few hundred native breeds can integrate and enhance the resources from the surrounding area, yet remain less productive, while intensive breeding of highly productive breeds is much more lucrative. Increased interest from the European Union and the consumer in environmental sustainability and biodiversity means that Lucanian breeders of native breeds need to supplement their income by making use of territorial resources, creating business diversifications which are all necessary conditions to help curb the abandonment of the territory. The importance of the presence of autochthonous breeds has also been highlighted by the survey which revealed the potential of this type of breeding, whilst also promoting cultural heritage to the younger generation. 

The knowledge system is needed to inform, educate and enable farms to improve their production methods, valorization and economic viability [41,42,43]. As such, some measures of the Basilicata RDP [44,45], such as information and consultancy services, must be made available to the agro-zootechnical system.

In European countries in the Mediterranean, sheep and goat farming is mainly practiced in marginal agricultural areas, these pastures very often represent the only form of productive use of the land and play an important role in preserving social, cultural and environmental values [46]. The INCIPIT project, “Start-up program for a conservation plan for the Altamurana sheep population”, assessed the economic sustainability of this kind of breeding which in the long-term resulted as economically unsustainable, as opposed to the Comisana allochthonous breed. The SHEEP UP project, funded by the Veneto region in 2019, aimed to “define an innovative model of integrated economic enhancement of sheep farming for indigenous breeds (Brogna, Foza, Lamon and Alpagota) in marginal areas of the Veneto mountains” [47].

The Lucanian biodiversity breeds are indigenous and rustic [20,48]; however, their product utilization is not capitalized as most of them are dual-purpose [48,49,50]. In order to maintain these breeds for the conservation of biodiversity it is essential to compensate lower income due to low productivity with adequate aid policies.

The role and importance of multifunctionality should not be underestimated, it can be achieved with complementary activities such as involving the entire family unit, allocating functions other than those specifically agricultural to better enhance products and their value [51,52]. Public policies are investing in actions capable of achieving the objectives of relaunching the sectors and seeking forms of support and improvement. Tools such as traceability, transparency, labeling, strengthening of the supply chain relationships, support for business diversification processes, promotion of short supply chains and direct sales of products processed on the farm, tourist incentives and the opening of rural areas and farms are among the clearest examples of new business models. Additional enabling factors must be considered to improve competitiveness, maintain biodiversity and to protect the territory and cultural heritage, particularly if located in marginal areas.

The protection of biodiversity is, and will increasingly be in the near future, vital to the consolidation of the productivity of any ecosystem. In order for this protection to become common practice, it is also necessary to provide adequate support for the protection of both vegetal and animal breed biodiversity, both in economic terms, and in terms of knowledge and services. The 2023–2037 Common Agricultural Policy (C.A.P.) [53] programming has paid due attention to the issue, providing financial instruments which play an important role in the knowledge system and support Lucanian farmers in the preservation of endangered breeds.

## 5. Conclusions

There is an increasing focus on the quality of agri-food products and as a result biodiversity breeding in Basilicata has an important and invaluable role in the livestock supply chain. Breeding stocks are deteriorating and support from the rural development policy is urgently needed. Over the years, regional development policies have contributed to protecting and increasing the number of breeds and in some cases have been successful in reaching their goals (Capra di Potenza and Rossa Mediterranea). Opportunities must be created to increase farmers’ income through the diversification of agricultural activities and improve their skills, knowledge and ability to introduce innovations and communicate the value and quality of their products. The surveys highlighted the need for consultancy services, improved infrastructure and new networks between farmers. Further lines of investigation could include the study of the profitability of these farms, for example high-quality brand, in marginal and inner areas [54,55].

## Data Availability

The data presented in this study are available on request from the corresponding authors.
